# Elucidation of the whole carotenoid biosynthetic pathway of aphids at the gene level and arthropodal food chain involving aphids and the red dragonfly

**DOI:** 10.1186/s40850-021-00082-w

**Published:** 2021-06-02

**Authors:** Miho Takemura, Takashi Maoka, Takashi Koyanagi, Naoki Kawase, Ritsuo Nishida, Tsutomu Tsuchida, Mantaro Hironaka, Tetsuyuki Ueda, Norihiko Misawa

**Affiliations:** 1 Research Institute for Bioresources and Biotechnology, Ishikawa Prefectural University, 1-308 Suematsu, Nonoichi-shi, Ishikawa 921-8836 Japan; 2 Research Institute for Production Development, 15 Shimogamo-morimoto-cho, Sakyo-ku, Kyoto 606-0805 Japan; 3 Department of Food Science, Ishikawa Prefectural University, 1-308 Suematsu, Nonoichi-shi, Ishikawa 921-8836 Japan; 4 Minakuchi Kodomono-mori Nature Museum, 10 Kitanaiki, Minakuchi-cho, Koka-shi, Shiga 528-0051 Japan; 5 Emeritus Prof., Kyoto University, Yoshida-honmachi, Sakyo-ku, Kyoto 606-8501 Japan; 6 Faculty of Science, Academic Assembly, University of Toyama, 3190 Gofuku, Toyama, 930-8555 Japan; 7 Department of Production Science, Ishikawa Prefectural University, 1-308 Suematsu, Nonoichi-shi, Ishikawa 921-8836 Japan; 8 Emeritus Prof., Ishikawa Prefectural University, 1-308 Suematsu, Nonoichi-shi, Ishikawa 921-8836 Japan

**Keywords:** Carotenoid biosynthesis, Functional analysis, *Escherichia coli*, Aphids, *Acyrthosiphon pisum*, *Sympetrum frequens*, *Nephila clavata*, *Oxyopes sertatus*, *Coccinella septempunctata*

## Abstract

**Background:**

Aphids can be positioned as robust pest insects in farming and as ones of the model organisms for arthropods in molecular biology. Carotenoids are pigments that protect organisms from photooxidative damage caused by excessive light. Aphids were shown to possess genes of fungal origin for carotenoid biosynthesis, whereas a little knowledge was available about the functions of the genes and the biosynthetic pathway. Even carotenoid species contained in aphids were not enough understood. Main purpose of this study is to clarify these insufficient findings.

**Results:**

The whole carotenoid biosynthetic pathway of the pea aphid (*Acyrthosiphon pisum*) was elucidated at the gene level, through comprehensive functional analysis of its carotenogenic genes, using *Escherichia coli* that synthesized carotenoid substrates, along with structural and quantitative analysis of carotenoids from various aphid species. Four genes were needed to synthesize all carotenoids accumulated in aphids from geranylgeranyl diphosphate. The *tor* gene mediated desaturation reaction from phytoene to 3,4-didehydrolycopene. It was revealed that a gene designated *ApCrtYB3*, which was considered to have functionally evolved in aphids, can convert lycopene into uncommon carotenoids with the γ-ring such as (6′*S*)-β,γ-carotene and γ,γ-carotene. We further demonstrated that the atypical carotenoids work as ecological indicators for estimating the food chain from aphids to predatory arthropods, and showed that aphids contributed with significant levels to the food chain from insect herbivores to several predatory arthropods, i.e., the red dragonfly (*Sympetrum frequens*; adults), seven-spotted ladybird (*Coccinella septempunctata*), and two spiders, *Oxyopes sertatus* and *Nephila clavata*. Gut microflora of the dragonfly (mature adults) was also found to include endosymbiotic bacteria such as *Serratia symbiotica* specific to the black bean aphid (*Aphis fabae*).

**Conclusions:**

We revealed the whole carotenoid biosynthetic pathway of aphids, including functional identification of the corresponding genes. Subsequently, we showed that arthropodal food chain can be estimated using the uncommon carotenoids of aphids as ecological indicators. This result indicated that aphids made significant contributions to the food chain of several predatory arthropods including the red-dragonfly adults. Aphids are likely to be positioned as an important “phytochemicals” source for some predatory insects and arachnids, which are often active under bright sunlight.

**Supplementary Information:**

The online version contains supplementary material available at 10.1186/s40850-021-00082-w.

## Background

Carotenoids are isoprenoid pigments with long conjugated double bonds, and exert many important physiological functions in organisms., e.g. as for their crucial functions, the protection of organisms from photooxidative damage caused by excessive light, and light harvest for photosynthesis [1, 2]. Carotenoid pigments are biosynthesized (synthesized de novo) not only in photosynthetic organisms such as higher plants and algae, but also in some species of bacteria, archaea and fungi [3–5]. On the other hand, animals (the kingdom Animalia) ordinarily cannot de novo synthesize carotenoids, and utilize exogeneous carotenoids acquired from their diet for supporting their health or life. However, several groups of arthropods (the phylum Arthropoda), which belong to the class Insecta or Arachnida, have unexpectedly been shown to possess carotenoid biosynthesis genes since 2010, i.e., they contain the pea aphid *Acyrthosiphon pisum* (order Hemiptera: family Aphididae) (class Insecta) [6], the two spotted spider mite *Tetranychus urticae* (Acari: Tetranychidae) (class Arachnida) [7], and the goldenrod gall midge (*Asteromyia carbonifera*) and the Hessian fly (*Mayetiola destructor*), flies of the family Cecidomyiidae (order Diptera) [8]. They commonly retained lycopene (carotene) β-cyclase/phytoene synthase (*CrtYB*) and phytoene desaturase (*CrtI*) genes, which are thought to have been acquired with lateral gene transfer from fungal donors and occasionally with subsequent differentiation [6–10]. A part of carotenogenic genes have been functionally analyzed in aphids [11, 12] and spider mites [13, 14]. As for aphids, a gene (named *tor*) [6] encoding carotene dehydrogenase was characterized to be significantly up-regulated in the red morph of the pea aphid [11], while its whole carotenoid biosynthetic pathway has remained unclear. Aphids that suck the sap of higher plants can be positioned as robust pest insects in farming, and further as ones of the model organisms for arthropods (animals) in molecular biology. Thus, we here aimed at elucidation of whole carotenoid biosynthetic pathway in aphids at the gene level.

Among the carotenogenic arthropods, the first carotenoid analysis was performed in spider mites and aphids more than 3.5 decades ago. Spider mite *T. urticae* that feeds on higher-plant cell contents was described to possess typical higher plant-type carotenoids such as β-carotene (β,β-carotene), lutein and neoxanthin, in addition to ketocarotenoids such as astaxanthin [15, 16]. As for aphids, the ordinary green morph of *Macrosiphum liliodendri* (family Aphididae) was reported to retain not only β-carotene but also carotenes with the γ-end group (the γ-ring) such as β,γ-carotene and γ,γ-carotene [16, 17] (called here “γ-carotenoids”), which are uncommon carotenoids in carotenogenic organisms [3] [attention, γ-carotenoids are entirely distinct from γ-carotene (β,ψ-carotene)]. However, no reports have described the presence of γ-carotenoids, β,γ-carotene and γ,γ-carotene, in aphids after the above-mentioned work. Instead, α-carotene (β,ε-carotene) and other carotenoids with the ε-ring such as δ-carotene (ε,ψ-carotene) have been described to exist in the green morph of aphids such as the pea aphid [6, 9, 18, 19]. Another purpose of this study is to clarify such an ambiguity on carotenoid species that aphids possess. On the other hand, the red aphid morph has been elucidated to additionally generate two red carotenoids, torulene (3′,4′-didehydro-β,ψ-carotene) and 3,4-didehydrolycopene (3,4-didehydro-ψ,ψ-carotene) [3, 6, 16, 17].

**Fig. 1:**
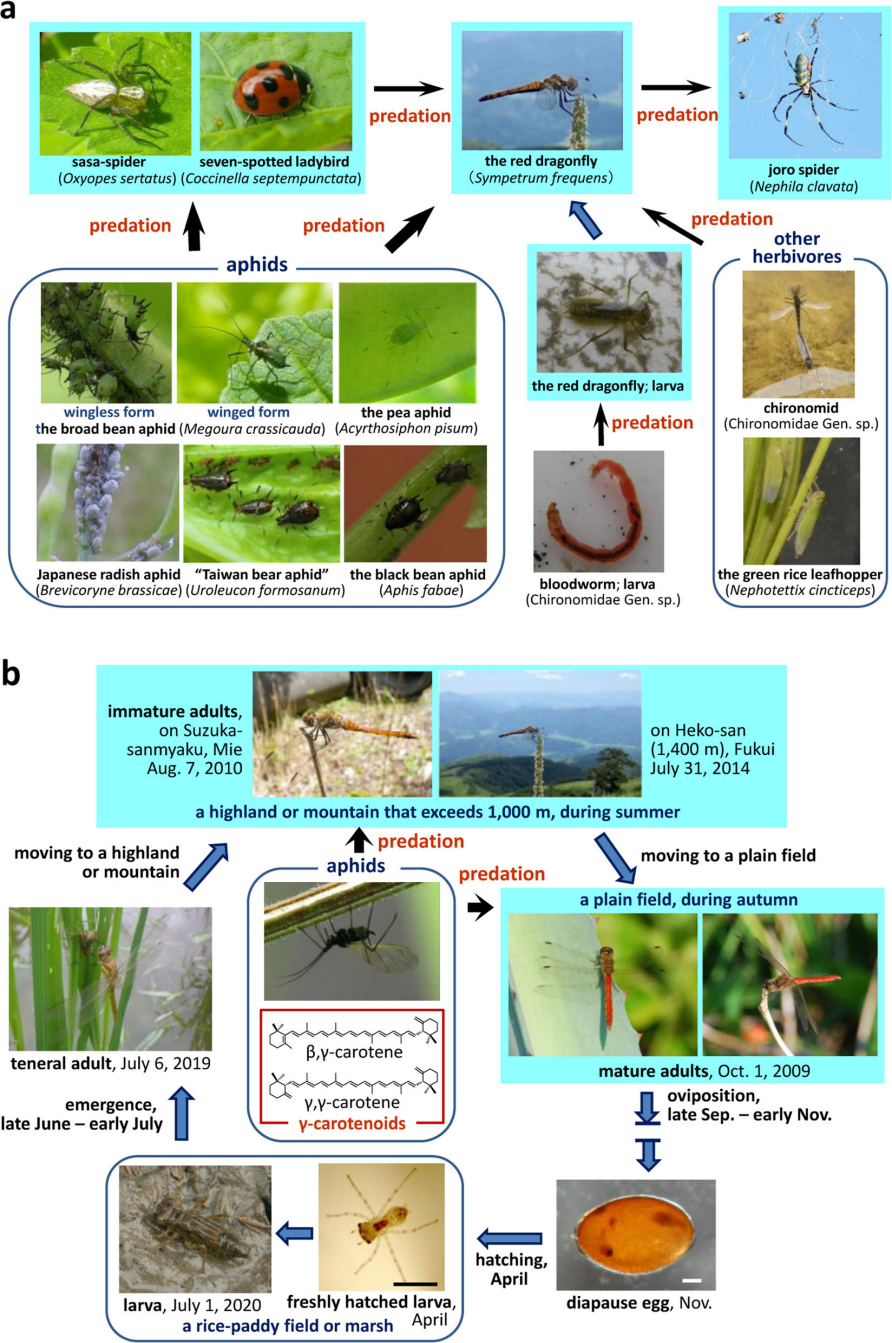
Arthropodal food chain, seen from the present study. **a)** Pictures of representative aphids related to this study, and food relationship among aphids, other herbivorous insects, and predatory arthropods. **b)** Life cycle of “akatombo” (the red dragonfly; *Sympetrum frequens*), and its food chain involving aphids. Information related to the individual shot images of Fig. 1 are described in Supplementary Note 1

If aphids truly acquire the ability to biosynthesize γ-carotenoids commonly, we can expect that γ-carotenoids may serve as ecological indicators on food relationship between aphids and other arthropods (Fig. 1a). A past work had also shown that seven-spotted ladybird *Coccinella septempunctata* (Coleoptera: Coccinellidae), which is well known to prey on aphids, accumulated γ-carotenoids [20]. *Sympetrum frequens* (Selys, 1883) (Odonata: Libellulidae), commonly known as “akatombo” in Japan, which means the red dragonfly, has been developed along with the long history of Japanese wet-paddy rice agriculture [21, 22]. Its unique life cycle is well known, as illustrated in Fig. 1b. Recently, Maoka et al. [23] conducted comprehensive carotenoid analysis on 20 dragonfly species (adults) including the red dragonfly, and consequently showed that they all possessed γ-carotenoids.

We here elucidate the whole carotenoid biosynthetic pathway of aphids at the gene level. We subsequently show that arthropodal food chain can be estimated using γ-carotenoids that were here confirmed to be biosynthesized in aphids, as ecological indicators, and consequently show that aphids are likely to make significant contribution to the food chain from insect herbivores to the red-dragonfly adults and other predatory arthropods. Gut microflora of the dragonfly is also analyzed to find aphid-specific endosymbiotic bacteria.

## Results

### Carotenoid analysis of aphids

Up to the present, carotenoid analysis of aphids has been conducted with various aphid species that include the pea aphid (*Acyrthosiphon pisum*) [6, 9, 16–19]. However, unexpectedly, there have been no reports showing the presence of γ-carotenoids, β,γ-carotene and γ,γ-carotene, except for the pioneering work shown above [16, 17]. Thus, we selected eight species of aphids (wingless form) belonging to the Aphididae family, which included *Megoura crassicauda* (ordinary green morph), *A. pisum* (ordinary green morph), *Brevicoryne brassicae*, *Uroleucon formosanum*, their pictures shown in Fig. 1a, and analyzed the carotenoids of these aphids quantitatively, along with their strict structural determination. Carotenoids were extracted from the eight aphid species, and were confirmed by their purification followed by spectroscopic analysis as shown in the Materials section, except for γ,ψ-carotene, which was unstable for its purification. The result is shown in Table 1. It was interestingly found that large chestnut aphid (*Lachnus tropicalis*) produced no carotenoids. The other seven aphid species commonly retained dominant amounts of β-carotene (β,β-carotene) and (6′*S*)-β,γ-carotene. γ-Carotene (β,ψ-carotene), γ,γ-carotene, and γ,ψ-carotene were often present in the aphids. β-Zeacarotene (7′,8′-dihydro-β,ψ-carotene) and torulene occasionally existed there. The chemical structures of these carotenoids are available in Fig. 3. α-Carotene (β,ε-carotene) and any other carotenoids with the ε-ring were not detected in the aphid samples we examined, although α-carotene has been reported to exist in aphids [6, 9, 18, 19]. It is possible that the carotenoid identified as α-carotene there was really β,γ-carotene, since α-carotene and β,γ-carotene are hardly distinguishable from each other in the retention time of high-performance liquid chromatography (HPLC), and UV-visible (UV-VIS) spectral and mass spectrometric (MS) data [3].

**Table 1:** Carotenoid content and composition of various aphid species that belong to the Aphididae family

Aphid species	The broad bean aphid	The pea aphid	Japanese radish aphid	“Taiwan bear aphid”	Celery aphid	The Asian woolly hackberry aphid	Cotton aphid	Large chestnut aphid
	*Megoura crassicauda*	*Acyrthosiphon pisum*	*Brevicoryne brassicae*	*Uroleucon formosanum*	*Semiaphis heraclei*	*Shivaphis celti*	*Aphis gossypii*	*Lachnus tropicalis*
Collected place^a^	Koka-shi, Shiga;	Koka-shi, Shiga;	Sakyo-ku, Kyoto	Sakyo-ku, Kyoto	Sakyo-ku, Kyoto	Sakyo-ku, Kyoto	Sakyo-ku, Kyoto	Sakyo-ku, Kyoto
	Sakyo-ku, Kyoto	Sakyo-ku, Kyoto						
Total carotenoid content (μg/g)	133.6	39.7	52.0	72.7	170	26.5	192.7	0
Carotenoid composition (%)								
β,β-carotene (β-carotene)	19.5	70.2	14.0	19.4	22.1	5.8	14.7	
β-zeacarotene					1.8	51.6	0.5	
β,ψ-carotene (γ-carotene)		2.2		33.5	11.2	13.5	58.5	
γ,ψ-carotene	13.7	1.2	12.0		7.5	6.4		
β,γ-carotene	48.8	15.9	33.8	38.8	33.7	14.8	23.6	
γ,γ-carotene	4.3	3.7	10.0		13.1			
Torulene			12.8					
Others	13.7	6.8	17.4	8.3	10.6	7.9	2.7	
Body number examined	50	100	45	56	45	50	not counted	not counted

^a^Aphid samples, whose all were wingless forms, were collected between May and June, 2019

### Functional analysis of carotenoid biosynthesis genes from the pea aphid using *E. coli*

In order to elucidate whole carotenoid biosynthetic pathway in aphids at the gene level, we performed functional analysis of all carotenogenic gene sequences that exist in the genome of the pea aphid (*A. pisum*). Primers were designed based on genome information on *A. pisum* (Supplementary Tables 1 and 2), and reverse transcription-polymerase chain reaction (RT-PCR) was carried out using total RNA isolated from the aphid. As a result, three and four distinct gene sequences were isolated, which were homologous to fungal genes, phytoene desaturase (*CrtI*) [24, 25] and lycopene (carotene) β-cyclase/phytoene synthase (*CrtY/CrtB*; *CrtYB*) [26], respectively, and designated *ApCrtI1–3* and *ApCrtYB1–4*. The *ApTor* (*tor*) gene that was reported to be involved in torulene biosynthesis [6, 11] was also homologous to *CrtI*, and thus was artificially synthesized, called *ApCrtI4* here. The nucleotide sequences of these genes are available under the accession numbers shown in Supplementary Table 1.

**Fig. 2:**
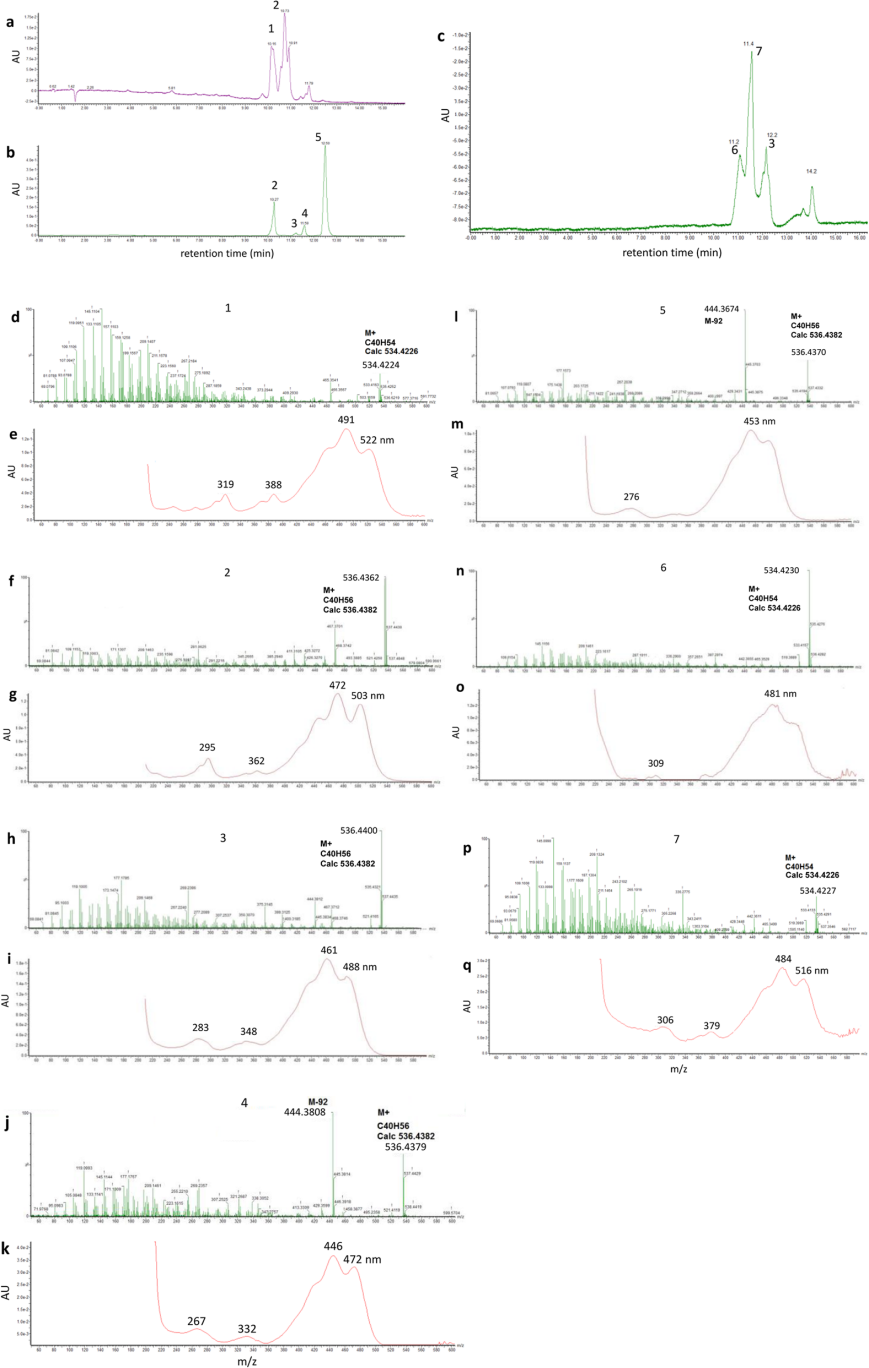
Functional analysis of the *ApCrtI4* (*ApTor*), *ApCrtYB3*, and *ApCrtYB1* genes. UPLC-PDA chromatograms (at 450 nm) of extracts from *E. coli* that carried pACHP-Phy plus pUC-ApTor (**a**), pAC-HIEBI-ApCrtYB3 (**b**), and pAC-HIEBI-ApCrtYB1 (**c**). Spectra of MS/MS (**d**) and UV-visible (**e**) of the peak **1** of **a** (3,4-didehydrolycopene), spectra of MS/MS (**f**) and UV-visible (**g**) of the peak **2** of **a** (lycopene), spectra of MS/MS (**h**) and UV-visible (**i**) of the peak **3** of **b** (γ-carotene), spectra of MS/MS (**j**) and UV-visible (**k**) of the peak **4** of **b** (β,γ-carotene), spectra of MS/MS (**l**) and UV-visible (**m**) of the peak **5** of **b** (β-carotene), spectra of MS/MS (**n**) and UV-visible (**o**) of the peak **6** of **c** (*cis*-torulene), and spectra of MS/MS (**p**) and UV-visible (**q**) of the peak **7** of **c** (torulene). **1**, 3,4-didehydrolycopene; **2**, lycopene; **3**, γ-carotene; **4**, β,γ-carotene; **5**, β-carotene; **6**, *cis*-torulene; **7**, torulene

Each of the *ApCrtI1–3* genes (plasmids pUC-ApCrtI1–3) was introduced into *E. coli* that synthesized phytoene (15-*cis*-phytoene) due to the presence of plasmid pACHP-Phy (see Supplementary Figure 3 for each plasmid). It was consequently found out that only ApCrtI2 (the *ApCrtI2* product) was able to accept phytoene as the substrate, and converted it to lycopene (peak 2) by way of neurosporene (peak 3) (Supplementary Figure 1). Thus, the *ApCrtI2* gene was identified to code for phytoene desaturase. When each of the *ApCrtYB1–4* genes (plasmids pUC-ApCrtYB1–4) was introduced into *E. coli* carrying plasmid pACHP-GGPP, which synthesized geranylgeranyl diphosphate (GGPP), only ApCrtYB3 synthesized phytoene (Supplementary Figure 2, peak 1). ApCrtYB3 was further shown to produce β-carotene (peak 4) from lycopene in the lycopene-producing *E. coli* (pACHP-Lyc) (Supplementary Figure 2). Thus, the *ApCrtYB3* gene proved to code for lycopene (carotene) β-cyclase/phytoene synthase. As shown in Fig. 2a, we also observed that the introduction of the *ApTor* (*ApCrtI4*) gene (plasmid pUC-ApTor) into the phytoene-producing *E. coli* (pACHP-phy) resulted in the synthesis of lycopene (peak 2; Fig. 2 fg) in addition to the carotenoid of peak 1, which was identified as 3,4-didehydrolycopene by its spectral data (Fig. 2de). It was therefore disclosed that the *ApTor* (*ApCrtI4*) gene encodes phytoene desaturase, which catalyzes 5-step dehydrogenation reactions from phytoene. This function of *ApCrtI4* was same as that of phytoene desaturase encoded by the *al-1* gene of the filamentous fungus *Neurospora crassa* [27].

**Fig. 3:**
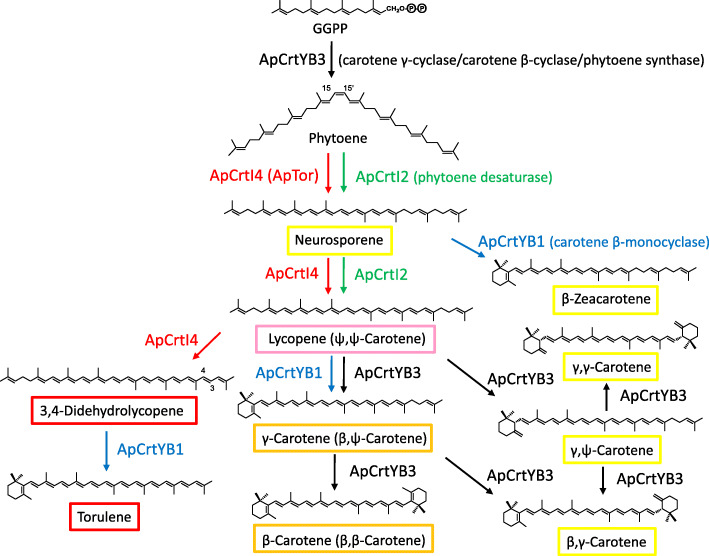
The whole carotenoid biosynthetic pathway of the pea aphid (*Acyrthosiphon pisum*) elucidated at the gene level. The coloured boxes represent typical colours of carotenoids surrounded by them

In order to carry out further functional analysis of the *ApCrtYB1–4* genes, we constructed other plasmids pAC-HIEBI-ApCrtYB1–4, respectively. Among these four plasmids, not only pAC-HIEBI-ApCrtYB3 but also pAC-HIEBI-ApCrtYB1 were found out to confer activity to metabolize lycopene on *E. coli*. Figure 2b revealed that *E. coli* (pAC-HIEBI-ApCrtYB3) biosynthesized the carotenoid of peak 4, which was thought to be β,γ-carotene by its spectral data (Fig. 2jk), in addition to β-carotene (peak 5; Fig. 2 lm) and γ-carotene (β,ψ-carotene; peak 3; Fig. 2hi). Further spectroscopic analysis confirmed the carotenoid (peak 4) to be (6′*S*)-β,γ-carotene as shown in the Methods section. It was revealed from these results that ApCrtYB3 acquired the ability to exert catalytic activity as carotene (lycopene) γ-cyclase, as well as carotene (lycopene) β-cyclase/phytoene synthase. Figure 2c showed that *E. coli* (pAC-HIEBI-ApCrtYB1) was able to biosynthesize the carotenoid of peak 7, which was identified to be torulene (3′,4′-didehydro-β,ψ-carotene) by its spectral data (Fig. 2pq), in addition to γ-carotene (peak 3). *cis*-Torulene (peak 6; Fig. 2no) was thought to be non-enzymatically converted from torulene. The *crtI* gene of *Pantoea ananatis* (reclassified from *Erwinia uredovora*) belonging to the γ-Proteobacteria class, which is included in plasmid pAC-HIEBI, can also mediate the synthesis of 3,4-didehydrolycopene from phytoene by way of lycopene [28–30]. It was thus considered that ApCrtYB1 converted lycopene and 3,4-didehydrolycopene into γ-carotene and torulene, respectively, indicating that the *ApCrtYB1* gene codes for carotene β-monocyclase. Through these comprehensive studies, the whole carotenoid biosynthetic pathway of the pea aphid was clarified at the gene level, as show in Fig. 3.

### Bacterial microbiota in feces of the red dragonfly

**Fig. 4:**
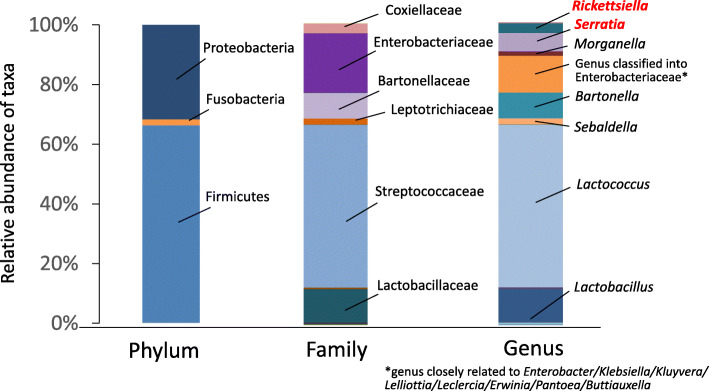
Bacterial microbiota in feces of the red dragonfly (*Sympetrum frequens*) at the phylum, family, and genus levels. Each taxon name with the existing ratio more than 1% was indicated in this figure. Bacteria species name reported as endosymbionts of aphids was described with red letter. Note that the feces from 30 to 50 dragonfly individuals were mixed to collect sufficient amounts, so the result shows averaged bacterial microbiota of multiple individuals (percentage of each taxonomic group represents an average in bacterial flora of multiple dragonflies)

In order to evaluate the gut microbiota of the red dragonfly (*Sympetrum frequens*), we carried out 16S rDNA amplicon analysis using high-throughput sequencing apparatus Illumina MiSeq as follows: Fresh feces (excrements) were collected from the living mature adults of *S. frequens*, and 16S rDNA V4 hyper-variable region was targeted to assess its bacterial microbiota structure (Fig. 4). At the phylum level, Proteobacteria (32% of total bacterial flora) and Firmicutes (66%) accounted for most of the gut microbiota of *S. frequens*. At the family/genus levels, it should be noted that lactic acid bacteria Streptococcaceae (*Lactococcus*; 54% of total bacterial flora) and Lactobacillaceae (*Lactobacillus*; 11%) were major constituents in the Firmicutes composition of the *S. frequens* feces. Multispecies of Proteobacteria were grouped into family Enterobacteriaceae (taxon closely related to *Enterobacter/Klebsiella/Kluyvera/Lelliottia/Leclercia/Erwinia/Pantoea/Buttiauxella* (12%), *Serratia* (6.1%) and *Morganella* (1.4%), Bartonellaceae (*Bartonella*; 8.5%), and Coxiellaceae (closely related to *Rickettsiella*, 3.0%). Other genera were less than 1% population in the bacterial microbiota. Among the above taxa, several bacteria belonging to the family Enterobacteriaceae, e.g. genera *Enterobacter* and *Erwinia/Pantoea*, can produce zeaxanthin and its derivatives such as zeaxanthin β-diglycoside [28, 31–33], thus these bacteria cannot accumulate a large part of carotenoids contained in the red-dragonfly mature adults (Table 2). β-Carotene producer *Pseudomonas* sp. was isolated from the feces of *S. frequens* in our recent study [34], but this genus was quite minor (not more than 0.19% of total bacteria). This bacterial strain is therefore unlikely to contribute to significant accumulation of β-carotene in *S. frequens* (Table 2). It is thus strongly suggested that carotenoid acquisition by *S. frequens* relies not on supply from gut microbes, but on oral food intake.

**Table 2:** Carotenoid content and composition of each stage in the life cycle of “akatombo” (the red dragonfly; *Sympetrum frequens*), and joro spider (*Nephila clavata*)

Arthropod species	The red dragonfly *Sympetrum frequens*				Joro spider
	Larvae	Teneral adults	Immature adults	Mature adults	*Nephila clavata*
Collected month	June, 2017	June, 2017	July, 2017	October, 2017	October, 2015
Collected place	in rice-paddy fields	in rice-paddy fields	on Mt. Gozaisyo-dake	near rice-paddy fields	near rice-paddy fields
	Koka-shi, Shiga	Koka-shi, Shiga	Komomo-cyo, Mie	Koka-shi, Shiga	Koka-shi, Shiga
Total carotenoid content (μg/g)	0.7	0.8	2.1	18.1	13.8
Carotenoid composition (%)					
β,β-carotene (β-carotene)	40.1	46.2	59.6	37.9	5.1
β-zeacarotene			2.5	10.1	1.3
β,ψ-carotene (γ-carotene)			7.8	8.3	8.1
γ,ψ-carotene					
β,γ-carotene			3.7	16.5	0.6
γ,γ-carotene			1.3	1.3	1.3
Torulene			1.5	2.2	1.7
β-caroten-2-ol	2.2	trace	3.8	4.2	2.9
Echinenone	1.8	3.2	4.2	3.5	5.2
Canthaxanthin	2.1				10.8
β-cryptoxanthin	5.2	26.8	9.5	5.6	4
Zeaxanthin		16.5	1.8	2.6	4.4
Lutein	14.2	4.5	3.4	4.0	21.7
Fucoxanthin	32.1				
Adonirubin^a^					12.3
Astaxanthin^b^					1.9
Others	2.3	2.8	0.9	3.8	18.7^c^
Body number examined	100	34	34	184	4

^a^ *R*: *S* (2:1)

^b^ *RR*: meso: *SS* (2:1:1)

^c^ other ketocarotenoids

It is very interesting that the closest species of detected genus *Serratia* was classified as *Serratia symbiotica* (100% identity over the V4 region, approximately 250 bp), which was reported as the secondary endosymbiont of the black bean aphid (*Aphis fabae*) [35], its picture shown in Fig. 1a. Intriguingly, γ-proteobacterium *Rickettsiella* was also reported to be the facultative endosymbiont of aphids [36, 37]. It is therefore very likely that gut microbiota of the red-dragonfly adults is reflecting the specific eating habit of the hosts, and that their carotenoid acquisition from aphids is the case.

### Carotenoid analysis of the red dragonfly and other arthropods

We carried out quantitative carotenoid analysis on individual life cycle stages of the red dragonfly (*S. frequens*), using large amounts of their samples (Table 2, Fig. 1b), while a similar carotenoid analysis was conducted with small amounts of the samples [23]. Quantitative analysis of carotenoids was further performed on the larvae and adults of seven-spotted ladybird (*Coccinella septempunctata*) (Supplementary Table 4), and two spiders *Oxyopes sertatus* (Araneae: Oxyopidae) (Supplementary Table 4) and *Nephila clavata* (Araneae: Araneidae) (Table 2), as predatory arthropods (Fig. 1a), and on the larvae (called bloodworm) and adults (called chironomid) of Chironomidae Gen. sp. (Diptera: Chironomidae), and the green rice leafhopper *Nephotettix cincticeps* (Hemiptera: Cicadellidae), as insect herbivores (Supplementary Table 3, Fig. 1a).

Interestingly, joro spider (*N. clavata*) was found to accumulate astaxanthin, adonirubin, and other ketocarotenoids, as shown in Table 2. It was recently revealed that the endogenous *CYP384A1* gene of spider mite *Tetranychus urticae*, which had evolved convergently, is very likely to code for carotenoid ketolase that synthesizes ketocarotenoids such as astaxanthin from β-carotene [14]. Joro spider may also retain such a cytochrome P450 gene, since many of its preys are unlikely to accumulate astaxanthin [3, 16].

## Discussion

This study reveals the whole carotenoid biosynthetic pathway that the pea aphid retains, including functional identification of the corresponding genes. (Fig. 3). Four genes are needed to synthesize all carotenoids accumulated in aphids from GGPP. They are *ApCrtI2* and *ApCrtI4* (also called *ApTor* or *tor* [6, 11]) as phytoene desaturase genes, and *ApCrtYB1* and *ApCrtYB3* as carotene cyclase genes. *ApCrtYB3* also codes for phytoene synthase. In the red aphid morph, ApCrtI4 is likely to work with ApCrtYB1 to produce torulene. ApCrtYB3 can function as carotene γ-cyclase to synthesize (6′*S*)-β,γ-carotene, γ,γ-carotene, and γ,ψ-carotene. Although the aphid carotenogenic genes are originated from fungi [6], the microbes ordinarily cannot produce such carotenoids with the γ-ring (called here γ-carotenoids). Only the discomycete fungus *Caloscypha fulgens* was shown to retain β,γ-carotene as a minor carotenoid [3]. Except for this fungus and aphids, no carotenogenic organisms have been reported to produce γ-carotenoids to our knowledge. Thus, the *ApCrtYB3* gene is considered to have functionally evolved in aphids. This is the first report on a gene for synthesizing γ-carotenoids.

We considered that the uncommon γ-carotenoids, which were here ascertained to be synthesized de novo in aphids and widely exist there, can work as ecological indicators for estimating the food chain from aphids to predatory arthropods. The content rate of all the γ-carotenoids that exist in the seven carotenogenic aphids (Table 1) average 40.2%. We carried out quantitative carotenoid analysis on polyphagous predatory arthropods that seem to eat aphids (Fig. 1ab), and on the aphid-specific insect predator, seven-spotted ladybird (the seven-spot ladybird beetle), and further on the top-positioned arachnid in arthropodal food chain, joro spider that is likely to prey not on aphids but on dragonflies (Fig. 1a). As expected, they were found to accumulate β,γ-carotene and γ,γ-carotene (Table 2, Supplementary Table 4). Content rate and contribution rate (content rate per 40.2) of these γ-carotenoids in the individual arthropods are calculated as follows (shown as percentage, respectively): the immature adults of the red dragonfly (*S. frequens*) (5, 12.4%), the mature adults of the dragonfly (17.8, 44.3%), the larvae of the seven-spot ladybird beetle (*C. septempunctata*) (24.1, 60%), the adults of the beetle (24.7, 61.4%), sasa-spider **(***O. sertatus*) (11, 27.4%), and joro spider (*N. clavata*) (1.9, 4.7%). These rate values are thought at high levels, especially noteworthy about the contribution rate (44.3%) of the red-dragonfly mature adults. It is feasible that the red-dragonfly adults prey on the winged form of aphids from “aerial plankton” (also called “aeroplankton”) composed of small drifting insects that are present high in the air [38]. This contribution of aphids is also supported by our findings that the gut microflora of the red-dragonfly mature adults included endosymbionts specific to aphids. On the other hand, we also conducted quantitative carotenoid analysis on several arthropods that are unlikely to feed on aphids, i.e., the aquatic larvae and teneral adults of the red dragonfly (Fig. 1b), the aquatic larvae and terrestrial adults of Chironomidae Gen. sp., and the green rice leafhopper (Fig. 1a), and consequently confirmed that no γ-carotenoids are detected there (Table 2, Supplementary Table 3).

## Conclusions

We here revealed whole carotenoid biosynthetic pathway in aphids that are ones of the model organisms for arthropods (animals) in molecular biology, at the gene level, including structural determination of carotenoid species that aphids possess. Aphids are also robust pest insects in farming. Carotenoids protect organisms against excessive light. Thus, a drug company may be able to develop a mild aphid-specific pesticide against the aphid carotenoid biosynthesis enzymes such as CrtI, since they are distinct from higher-plant carotenogenic enzymes.

Subsequently, we demonstrated that estimation of arthropodal food chain was feasible using the uncommon carotenoids, γ-carotenoids, synthesized de novo in aphids, as ecological indicators. The result indicated that aphids made significant contributions to the food chain from insect herbivores to the red-dragonfly adults and other predatory arthropods. Aphids are likely to be positioned as an important “phytochemicals” source not only for aphid-specific insect predators, but also for some polyphagous predatory insects and arachnids, which are often active under bright sunlight.

## Methods

### Arthropods

As aphid materials we used the broad been aphid (*Megoura crassicauda*; green morph), the pea aphid (*Acyrthosiphon pisum*; green morph), Japanese radish aphid (cabbage aphid; *Brevicoryne brassicae*), “Taiwan bear aphid” (Formosan hairy aphid; *Uroleucon formosanum*), celery aphid (*Semiaphis heraclei*), the Asian woolly hackberry aphid (*Shivaphis celti*), cotton aphid (*Aphis gossypii*), and large chestnut aphid (*Lachnus tropicalis*) (their wingless forms; Table 1). As the other arthropod materials we used “akatombo” (the red dragonfly; *Sympetrum frequens*) (its several developmental stages), joro spider (*Nephila clavata*; an Oriental species of golden orb-weaving spider) (Table 2), the larvae (bloodworms) and adults (chironomids) of Chironomidae Gen. sp., the green rice leafhopper (*Nephotettix cincticeps*) (Supplementary Table 3), sasa-spider (“sasa-gumo”; *Oxyopes sertatus*; an Oriental species of foraging spider), and the larvae and adults of seven-spotted ladybird (the seven-spot ladybird beetle; *Coccinella spetempunctata*) (Supplementary Table 4).

### Cloning of aphid carotenogenic genes

Total RNA was extracted from the pea aphids *A. pisum*, cDNA was synthesized by the reverse transcriptase ReverTra Ace (TOYOBO, Osaka, Japan), and used as the template for PCR. Based on its genome sequence (AphidBase, https://bipaa.genouest.org/is/aphidbase/), primers were designed as shown in Supplementary Table 1, and used to amplify the coding regions of individual carotenogenic genes by PCR, followed by their sequence confirmation. The obtained coding regions (PCR products) were digested with *Eco*RI and *Sal*I (or *Xho*I), and ligated with an *E. coli* vector pUC18 [ampicillin (Ap) resistance (Ap^r^); Takara Bio, Otsu, Japan] that was digested with *Eco*RI and *Sal*I. The *ApTor* (*ApCrtI4*) gene was chemically synthesized (Supplementary Table 1), and inserted into pUC18 same. Resultant plasmids were named pUC-ApCrtI1–3 (for *ApCrtI1–3*), pUC-ApCrtYB1–4 (for *ApCrtYB1–4*), and pUC-ApTor (for *ApTor*) (Supplementary Figure 3).

### Plasmid construction

Plasmids pACHP-Lyc (for lycopene synthesis), pACHP-Phy (for phytoene synthesis), and pACHP-GGPP (for GGPP synthesis) were constructed by disrupting *crtY*, *crtI* and *crtY*, and *crtB*, *crtI* and *crtY*, respectively, from plasmid pACHP-Beta [chloramphenicol (Cm) resistance (Cm^r^)] that synthesizes β-carotene due to the presence of the *Haematococcus pluvialis IDI* [isopentenyl diphosphate (IPP) isomerase], *Pantoea ananatis crtE* (GGPP sytnthase), *crtB* (phytoene synthase), and *crtI* (phytoene desaturase), and *crtY* (lycopene β-cyclase) genes [39–41].

We further constructed a plasmid named pAC-HIEBI for lycopene synthesis, which contained the *H. pluvialis IDI*, *P. ananatis crtE*, *crtB*, and *crtI* genes, flanked by the *tac* promoter (P*tac*) and the *rrnB* terminator (T*rrnB*), as follows: P*tac* and T*rrnB* fragments were amplified by PCR using primer sets, PtacF and PtacR, and TrrnBF and TrrnBR (Supplementary Table 2), respectively. The amplified P*tac* and T*rrnB* fragments were inserted into the *Eco*RI and *Nco*I sites of an *E. coli* vector pACYC184 [Cm and tetracycline (Tc) resistance (Tc^r^)] [42], resulting in the disruption of the Cm resistance gene of the vector. Next, the *IDI*, *crtE*, *crtB*, and *crtI* gene fragments were amplified by PCR using primer sets, HpIDIF and HpIDIR, CrtEF and CrtER, CrtBF and CrtBR, and CrtIF and CrtIR, respectively. These fragments were together ligated, and inserted into the *Xho*I and *Kpn*I sites between P*tac* and T*rrnB* of the above-constructed plasmid, yielding plasmid pAC-HIEBI. The coding regions of *ApCrtYB1–4* were amplified by PCR using the corresponding primers as shown in Supplementary Table 2. The PCR fragments were digested with *Bam*HI and *Kpn*I, and inserted into the *Bgl*II and *Kpn*I site of pAC-HIEBI. The resultant plasmids were named pAC-HIEBI-ApCrtYB1–4 (for *ApCrtYB1–4*). The structures of the constructed plasmids are shown in Supplementary Figure 3.

### Culture of *E. coli*

The constructed plasmids were introduced into *E. coli* strain JM101 (DE3) [43]. The transformed *E. coli* cells were cultured in 2YT medium (1.6% Bactotryptone, 1% yeast extract, 0.5% NaCl) containing Ap (40 mg/L) and Cm (30 mg/L), or Tc (10 mg/L) at 37 °C. Then, we inoculated this preculture into the new 2YT medium with the same antibiotic(s) and 0.05 mM isopropyl-β-D-thiogalactopyranoside (IPTG), and cultured at 20 °C for 2 days.

### Analysis of carotenoids

As methods for *E. coli*, which include those in Supplementary Figures 1 and 2, we extracted and analyzed carotenoids as described [44]. The *E. coli* cultures were centrifuged, and cell pellets were extracted with methanol (MeOH) using mixer for 5 min. Tris-HCl (50 mM, pH 7.5) and 1 M NaCl was added and mixed. Then, chloroform was added and mixed for 5 min. After centrifugation, the chloroform phase was collected and dried by centrifugal evaporation. Dried residues were re-suspended with ethyl acetate, and applied to HPLC with a Waters Alliance 2695–2996 (PDA) system (Waters, Milford, MA, USA). HPLC was carried out using TSKgel ODS-80Ts (4.6 × 150 mm, 5 μm; Tosoh, Tokyo, Japan). The crude extract was eluted at a flow rate of 1.0 ml/min at 25 °C with solvent A (water-MeOH, 5:95, v/v) for 5 min, followed by a linear gradient from solvent A to solvent B (tetrahydrofuran-MeOH, 3:7, v/v) for 5 min, and solvent B alone for 8 min. Individual carotenoids were identified by comparing retention times and absorption spectra with those of the authentic samples that were extracted from recombinant *E. coli* cells [28, 29].

As methods for *E. coli* in Figs. 1 and 2, and all arthropod samples that are described in Tables 1 and 2 and Supplementary Tables 3 and 4, we extracted and analyzed carotenoids according to similar methods to those described [23]. Pigments were extracted from the *E. coli* cells and the arthropod samples with acetone at room temperature, and then transferred to *n*-hexane: diethyl ether (Et_2_O) (1:1, v/v) by adding water. The *n*-hexane: Et_2_O phase was washed with water and dehydrated on anhydrous sodium sulphate. The total carotenoid amounts were calculated using coefficient of ${\mathrm{E}}_{\mathrm{cm}}^{1\%}$=2400 at λ max. Quantitative and qualitative carotenoid analysis of the extracted carotenoids was carried out as follows. The LC/MS analysis of carotenoids was carried out using a Waters Xevo G2S Q TOF mass spectrometer (Waters Corporation, Milford, CT, USA) equipped with an Acquity UPLC system. The electro-spray ionization (ESI) time-of-flight (TOF) MS spectra were acquired by scanning from *m/z* 100 to 1500 with a capillary voltage of 3.2 kV, cone voltage of 20 eV, and source temperature of 120 °C. Nitrogen was used as a nebulizing gas at a flow rate of 30 L/h. Tandem mass spectrometric (MS/MS) spectra were measured with a quadrupole-TOF MS/MS instrument with argon as a collision gas at a collision energy of 20 V. UV-VIS absorption spectra were recorded from 200 to 600 nm using a photodiode-array detector (PDA). An Acquity 1.7 μm BEH UPLC C18 (2.1 id X 100 mm) column (Waters Corporation, Milford, CT, USA) was used for the UPLC system, developed by acetonitrile (MeCN):H_2_O (85:15) - MeCN:MeOH (65:35) (linear gradient 0 to 15 min) as a mobile phase, at a flow rate of 0.4 mL/min. Carotenoids were identified by UV-VIS, MS, and MS/MS spectral data and retention time in HPLC (UPLC) with comparison of authentic samples that had been purified in the T. Maoka’s Lab.

As needed, e.g., in case of *E. coli* (pAC-HiEBI-ApCrtYB3) and several aphids, individual carotenoids were isolated by column chromatography followed by preparative HPLC, and identified from their UV-VIS, ESI TOF MS, ^1^H nuclear magnetic resonance (NMR), and circular dichroism (CD) spectral data. The UV-VIS spectra were recorded with a Hitachi U-2001 spectrophotometer (Hitachi Field Navigator, Tokyo, Japan) in Et_2_O. The ^1^H NMR (500 MHz) spectra were measured with a Varian UNITY INOVA 500 spectrometer (Varian Corporation, Palo Alto, California USA) in CDCl_3_ with TMS as an internal standard. The CD spectrum was recorded in Et_2_O at room temperature with a Jasco J-500C spectropolarimeter (JASCO Corporation, Hachioji, Tokyo, Japan). Preparative HPLC was performed with a Hitachi L-6000 intelligent pump and an L-4250 UV-VIS detector (Hitachi Field Navigator, Tokyo, Japan) set at 450 nm. The column used was a 250 X 10 mm i.d., 5 μm Cosmosil 5C_18_-MS-II ODS (Nacalai Tesque, Kyoto, Japan) with CHCl_3_:MeOH (25:75, v/v) as a solvent at a flow rate of 2.0 mL/min. The extracted carotenoids were separated by silica gel chromatography using *n*-hexane, Et_2_O, and acetone as eluting solvents. Carotenoids eluted with *n*-hexane was further separated by preparative ODS HPLC to afford β-carotene, (6′*S*)-β,γ-carotene, β-zeacarotene, β,ψ-carotene (γ-carotene), torulene, and γ,γ-carotene.

Spectroscopic data of (6′*S*)-β,γ-carotene and γ,γ-carotene are as follows, which were in agreement with published data [17, 23].

(6′*S*)-β,γ-Carotene: ESI TOF MS (*m/z*) 536.4379 [M^+^] C_40_H_56_, Calcd for 536.4382; UV-Vis (Et_2_O) 412, 442, 471 nm; ^1^H NMR (see Supplementary Note 2); CD (in Et_2_O): λ nm (Δε) 216 (+ 3.0), 230 (0), 238 (− 4.8), 249 (0), 270 (+ 4.6), 225 (0), 340 (− 1.8), 375 (− 0.2).

γ,γ-Carotene: ESI TOF MS (*m/z*) 536.4383 [M^+^] C_40_H_56_, Calcd for 536.4382; UV-Vis (Et_2_O) 419, 439, 468 nm; ^1^H NMR (see Supplementary Note 2).

### Gut microbiota analysis

Fresh feces (excrements) were collected using the living 30–50 individual dragonflies of *S. frequens* (mature adults), which were captured near rice-paddy field in Koka-shi, Shiga, in September, 2017, followed by bacterial genomic DNA extraction with EZ-Extract® for DNA (AMR Inc., Gifu, Japan) and a NucleoSpin® Tissue kit (Macherey-Nagel GmbH & Co. KG, Düren, Germany) according to the manufacturer’s instructions. The V4 hyper-variable region of 16S ribosomal RNA gene (16S rDNA) was amplified by PCR with PrimeSTAR® GXL DNA polymerase (Takara Bio, Otsu, Japan) and a specific primer pair (515F1 and 806R1; Supplementary Table 2) with 35 cycles of denaturation (98 °C, 10 s), annealing (50 °C, 15 s), and extension (68 °C, 30 s). After the checking of the appearance of single DNA bands on 2 (w/v)% agarose gel with correct size (approximately 300 bp), the fragments were purified by QIAquick® PCR purification kit (Qiagen, Venlo, the Netherlands). The 2nd PCR was performed using the same V4 primers but with overhanging Illumina MiSeq-specific P5/P7 adapters and 12 bp index sequences (515F2 and 806R2; Supplementary Table 2), with a limited-cycle PCR using the 1st PCR amplicon as a template. After purification by 2% agarose-gel electrophoresis and following extraction from the gel by using a Wizard® SV Gel and PCR Clean-Up System (Promega, Madison, WI), the paired-end sequencing was performed on an Illumina MiSeq instrument. Data obtained were processed using the QIIME pipeline (version 1.8) [45], where the non-chimeric reads were clustered into operational taxonomic units (OTUs) at a 97% cutoff threshold. The taxonomic classification of each OTU was carried out at the phylum, family, and genus levels by applying the sequence reads toward the Ribosomal Database Project (RDP) classifier program [46] with use of the Greengenes 16S rDNA database [47]. The closest species of representative sequences were identified by the Basic Local Alignment Search Tool (BLAST) (http://blast.ncbi.nlm.nih.gov/Blast.cgi).

## Supplementary Information

**Additional file 1: Supplementary Figure 1.** Functional analysis of three phytoene desaturase gene sequences (*ApCrtI1*, *ApCrtI2*, and *ApCrtI3*) existing in the genome of the pea aphid. **Supplementary Figure 2.** Functional analysis of four carotene (lycopene) β-cyclase/phytoene synthase gene sequences (*ApCrtYB1*, *ApCrtYB2*, *ApCrtYB3*, and *ApCrtYB4*) existing in the genome of the pea aphid. **Supplementary Figure 3.** Plasmids constructed and used in this study. **Supplementary Table 1.** Carotenoid biosynthesis genes of the pea aphid analyzed in this study and their primer sequences for PCR. **Supplementary Table 2.** The other primer sequences for PCR. **Supplementary Table 3.** Carotenoid content and composition of other insect herbivores. **Supplementary Table 4.** Carotenoid content and composition of predatory arthropods that eat aphids. **Supplementary Note 1.** Information related to the individual shot images in Fig. 1. **Supplementary Note 2.** ^1^H NMR spectral data of (6′*S*)-β,γ-carotene and γ,γ-carotene.

## Data Availability

The carotenogenic gene sequences of the pea aphids are deposited in the DNA Data Bank of Japan (DDBJ) under the accession numbers LC517091 -LC517098. The gut microbiota sequence data for the mature adults of the red dragonfly are available at the DDBJ DRA database as accession number DRA010417 under BioProject PRJDB10124, BioSample SAMD00233776. The other data generated and/or analyzed during this study are included in this published article and its supplementary information file.
