# Supplementary material for: Elucidation of the whole carotenoid biosynthetic pathway of aphids at the gene level and arthropodal food chain involving aphids and the red dragonfly
Source: BMC Zool. 2021 Jun 2;6:19. doi: 10.1186/s40850-021-00082-w (PMC10127341; doi:10.1186/s40850-021-00082-w)
Supplement: Supplementary file 1 — Additional file 1: Supplementary Figure 1. Functional analysis of three phytoene desaturase gene sequences (ApCrtI1, ApCrtI2, and ApCrtI3) existing in the genome of the pea aphid. Supplementary Figure 2. Functional analysis of four carotene (lycopene) β-cyclase/phytoene synthase gene sequences (ApCrtYB1, ApCrtYB2, ApCrtYB3, and ApCrtYB4) existing in the genome of the pea aphid. Supplementary Figure 3. Plasmids constructed and used in this study. Supplementary Table 1. Carotenoid biosynthesis genes of the pea aphid analyzed in this study and their primer sequences for PCR. Supplementary Table 2. The other primer sequences for PCR. Supplementary Table 3. Carotenoid content and composition of other insect herbivores. Supplementary Table 4. Carotenoid content and composition of predatory arthropods that eat aphids. Supplementary Note 1. Information related to the individual shot images in Fig. 1. Supplementary Note 2. 1H NMR spectral data of (6′S)-β,γ-carotene and γ,γ-carotene. [file 40850_2021_82_MOESM1_ESM.docx]

**<Supplementary Information>**

**Elucidation of the whole carotenoid biosynthetic pathway of aphids at the gene level and arthropodal food chain involving aphids and the red dragonfly**

Miho Takemura, Takashi Maoka, Takashi Koyanagi, Naoki Kawase, Ritsuo Nishida, Tsutomu Tsuchida, Mantaro Hironaka, Tetsuyuki Ueda, and Norihiko Misawa

**Supplementary Figure 1. Functional analysis of three phytoene desaturase gene sequences (*ApCrtI1*, *ApCrtI2*, and *ApCrtI3*) existing in the genome of the pea aphid.**

HPLC-PDA chromatograms of extracts from *E. coli* that carried pACHP-Phy plus pUC-ApCrtI1 (upper, *ApCrtI1*), pACHP-Phy plus pUC-ApCrtI2 (middle, *ApCrtI2*), and pACHP-Phy plus pUC-ApCrtI3 (lower, *ApCrtI3*). UV-visible spectra of peaks 1, 2, and 3 are shown in right column. 1, phytoene; 2, lycopene; 3, neurosporene.

**Supplementary Figure 2. Functional analysis of four carotene (lycopene) β-cyclase/phytoene synthase gene sequences (*ApCrtYB1*, *ApCrtYB2*, *ApCrtYB3*, and *ApCrtYB4*) existing in the genome of the pea aphid.**

HPLC-PDA chromatograms of extracts from *E. coli* that carried pACHP-GGPP plus pUC-ApCrtYB1-4 (left column, *ApCrtYB1-4*), and pACHP-Lyc plus pUC-ApCrtYB1-4 (middle column, *ApCrtYB1-4*). UV-visible spectra of peaks 1, 2, and 4 are shown in right column. 1, phytoene; 2, lycopene; 4, β-carotene.

**Supplementary Figure 3. Plasmids constructed and used in this study.**

*1, XXX indicates either CrtI1, CrtI2, CrtI3, CrtYB1, CrtYB2, CrtYB3, CrtYB4, or Tor, which carries the corresponding genes. *2, ApCrtYB represents either ApCrtYB1, ApCrtYB2, ApCrtYB3, or ApCrtYB4, which retains the corresponding gene.

**Supplementary Note 1. Information related to the individual shot images in Figure 1.**

**a)** Photo information on the individual aphids are as follows: the broad bean aphid (wingless form), in Nonoichi-shi, Ishikawa on May 16, 2020 by NM; the broad bean aphid (winged form),in Koka-shi, Shiga on May 25, 2018 by NK; the pea aphid, in Koka-shi on May 24, 2020 by NK; Japanese radish aphid, in Koka-shi on May 6, 2018 by NK; “Taiwan bear aphid”, in Koka-shi on June 3, 2018 by NK; the black bean aphid, in Nonoichi-shi on June 1, 2020 by NM. The pictures of the red-dragonfly larva, adult, and joro spider were taken in Koka-shi on June 11, 2015 by NK, in Fukui-shi on July 31, 2014 by TU, and in Yokosuka-shi, Kanagawa on Oct. 1, 2009 by Kiyoshi Hagiwara, respectively. The snapshots of sasa-spider and seven spotted ladybird were taken in Koka-shi by NK on May 14, 2018 and on Sep. 5, 2016, respectively. Photo information on other herbivorous insects are as follows: bloodworm, in Koka-shi on Sep. 8, 2009 by NK; chironomid, in Koka-shi on Feb. 22, 2016 by NK; the green rice leafhopper, in Hamamatsu-shi, Shizuoka on Jan. 22, 2015 by MH.

**b)** The pictures of diapause egg and freshly hatched larva were recorded by TU. The photos of larva (July), and teneral adult were taken in a rice-paddy field, Nonoichi-shi by Jun-ichiro Hattan, and in Minakuchi Kodomono-mori Museum, Koka-shi by NK, respectively. The pictures of immature adults (left and right) were recorded by NK and TU, respectively. The snapshots of the two mature adults were taken in Tenjin-jima, Yokosuka-shi by Kiyoshi Hagiwara. The picture of the winged form of the broad bean aphid is shown as one example of aphids, which was recorded in Ishikawa Prefectural University, Nonoichi-shi on May 13, 2020 by Jun-ichiro Hattan.

**Supplementary Note 2. ^1^H NMR spectral data of (6’*S*)-β,γ-carotene and γ,γ-carotene**

(6’*S*)-β,γ-Carotene: ^1^H NMR δ (in CDCl_3_ at 500 MHz) 0.82 (3H, s, H-16’), 0.90 (3H, s, H-17’), 1.03 (6H, s, H-16,17), 1.33 (2H, dd, *J*=14, 7 Hz, H-2’), ~1.46 (2H, m, H-2), ~1.63 (2H, m, H-3), 1.72 (3H, s, H-18), 1.95 (3H, s, H-19’), 1.97 (9H, s, H-19, 20, 20’), 2.02 (2H, t, *J*=7 Hz, H-4), 2.07 (2H, m, H-3’), 2.29 (2H, m, H-4’), 2.51 (1H, d, *J*=9.5 Hz, H-6’), 4.57 (1H, br. s, H-18’), 4.73 (1H, br. s, H-18’), 5.84 (1H, dd, *J*=15.5, 9.5 Hz, H-7’), 6.12 (1H, d, *J*=11.5 Hz, H-10’), 6.13 (1H, d, *J*=15.5 Hz, H-8’), 6.14 (1H, d, *J*=16 Hz, H-8), 6.14 (1H, d, *J*=10 Hz, H-10), 6.15 (1H, d, *J*=16 Hz, H-7), 6.24 (2H, m, H-14, 14’), 6.33 (1H, d, *J*=15.5 Hz, H-12’), 6.35 (1H, d, *J*=15.5 Hz, H-12), 6.62 (1H, dd, *J*=15.5, 11.5 Hz, H-11’), 6.63 (2H, m, H-15, 15’), 6.65 (1H, dd, *J*=15.5, 11.5 Hz, H-11’).

γ,γ-Carotene: ^1^H NMR δ (in CDCl_3_ at 500 MHz) 0.82 (6H, s, H-16, 16’), 0.90 (6H, s, H-17, 17’), 1.33 (4H, dd, *J*=14, 7 Hz, H-2, 2’, 1.95 (6H, s, H-19, 19’), 1.97 (6H, s, H-20, 20’), 2.07 (4H, m, H-3, 3’), 2.29 (4H, m, H-4, 4’), 2.51 (2H, d, *J*=9.5 Hz, H-6, 6’), 4.57 (2H, br. s, H-18, 18’), 4.73 (2H, br. s, H-18, 18’), 5.84 (2H, dd, *J*=15.5, 9.5 Hz, H-7, 7’), 6.12 (2H, d, *J*=11.5 Hz, H-10, 10’), 6.13 (2H, d, *J*=15.5 Hz, H-8, 8’), 6.24 (2H, m, H-14, 14’), 6.33 (2H, d, *J*=15.5 Hz, H-12, 12’), 6.61 (2H, dd, *J*=15.5, 11.5 Hz, H-11, 11’), 6.63 (2H, m, H-15, 15’).
